# YAP‐TEAD up‐regulates IRS2 expression to induce and deteriorate oesophageal cancer

**DOI:** 10.1111/jcmm.16266

**Published:** 2021-02-11

**Authors:** Xiangming Xu, Jiao Nie, Lin Lu, Chao Du, Fansheng Meng, Duannuo Song

**Affiliations:** ^1^ Department of Gastroenterology Linyi People's Hospital Linyi China

**Keywords:** IRS2, JNK/c‐Jun pathway, oesophageal cancer, TEAD, YAP

## Abstract

Oesophageal cancer (EC) represents a significant cause of cancer worldwide. Yes‐associated protein (YAP) is reported to correlate with the initiation of multiple cancers including EC, but the underlying mechanism remains elusive. The current study aimed to investigate the molecular mechanism of YAP‐TEAD in the occurrence and progression of EC. EC tissues and cells were obtained, followed by determination of the expression of YAP, c‐Jun, pc‐Jun and IRS2. The effect of YAP‐TEAD on the biological EC cell processes was explored through gain‐ and loss‐of‐function approaches. The interaction between YAP and TEAD was detected by co‐immunoprecipitation. The binding of TEAD to the c‐Jun promoter was determined using chromatin immunoprecipitation. Tumour formation in the nude mice was detected in order to ascertain the effect of YAP and IRS2 in vivo. We found elevated YAP in the EC tissues and cells. YAP silencing led to a decrease in EC cell proliferation, invasion and sphere formation. YAP‐TEAD complex bound to the promotor of c‐Jun, and c‐Jun led to an increase in the expression of IRS2 through the JNK/c‐Jun pathway. Additionally, pc‐Jun and phosphorylated JNK were localized in the nuclear in addition to displaying enhanced expression in the EC tissues. IRS2 overexpression negated the inhibition of cell proliferation, invasion and sphere formation triggering YAP silencing. YAP up‐regulated IRS2 and aggravated EC *in vivo*. Taken together, YAP‐TEAD activates the JNK/c‐Jun pathway to up‐regulate IRS2, ultimately promoting EC progression. Therefore, YAP‐TEAD inhibition could be a promising therapeutic approach for EC treatment.

## 1 INTRODUCTION

As a prevalent and aggressive cancer, oesophageal cancer (EC) can be subcategorized into two major histological forms, including adenocarcinoma (EAC) and squamous cell carcinoma (ESCC),^1^ the latter of which affects the thin cells on the surface of the oesophagus and accounts for approximately 90% of all the EC cases on a global scale.^2^ Although a wide variety of conventional EC therapy exists, including surgery, radiotherapy and chemotherapy,^3^ patient prognosis has stagnated highlighting insufficient knowledge in regard to the progression of ESCC. Thus, extending our understanding of EC on both a genetic and molecular level represents a crucial target for the continued development of future therapies.

Yes‐associated protein (YAP) was initially regarded as a protein associated with Yes, a src family kinase (SFK).^4^ Previous literature has emphasized the dysregulation of YAP function as a crucial driver of tumorigenesis, chemoresistance and metastasis.^5^ A previous study provided evidence of high levels of YAP expression in ESCC tissues.^6^ Additionally, the oncogenic activity of YAP has been shown to be mediated by the TEA Domain (TEAD) family transcription factors,^7^, ^8^, ^9^ which led us to further investigate its role in ESCC in vitro and in vivo. Aberrant expression of TEAD has been documented to influence well‐known cancer genes such as KRAS and BRAF, with its transcriptional output implicated in various processes including cancer metabolism, tumour progression and cancer metastasis.^10^ Existing literature has highlighted that the YAP/PDZ‐binding motif (TAZ)/TEAD interacts with AP‐1 to facilitate tumour growth,^11^ while inhibition of YAP/TAZ‐TEAD has recently emerged as a promising therapeutic target for various types of cancers.^4^ Interestingly, TEAD is involved in the promoting effects of YAP on EC.^12^

The c‐Jun N‐terminal kinase (JNK), a member of the mitogen‐activated protein kinase (MAPK) family, regulates both cancer cell apoptosis and survival.^13^ The aberrant activation of JNK has been shown to consequently result in the deterioration in different cancers, including oral,^14^ prostate^15^ and pancreatic cancer.^16^ Among the vast substrates of JNK, the oncogene c‐Jun stands out due to its strong association with cancer invasiveness.^17^ Considering the c‐Jun promoter contains TEAD binding site,^18^ we asserted the hypothesis that the JNK/c‐Jun pathway is regulated by YAP‐TEAD.

Yes‐associated protein has been previously reported to positively regulate insulin receptor substrate 2 (IRS2) to affect the activity of non‐small cell lung cancer cells,^19^ highlighting the relationship between YAP and IRS2. IRS2 represents a signalling molecule capable of mediating the effects of insulin/insulin‐like growth factor 1 (IGF1). IRS2 is expressed in various types of cancer and has been reported to contribute to tumour cell metabolism.^20^ Suppression of IRS2 has been shown to confer an inhibitory effect on the progression of liver cancer^21^ neuroblastoma^22^ and ESCC.^23^ Moreover, IRS2 is the target gene of the JNK/c‐Jun pathway in breast cancer cells.^24^

Here, we set out to determine whether YAP‐TEAD could induce and deteriorate ESCC by means of regulating IRS2 via the JNK/c‐Jun axis by conducting in vitro and in vivo assays.

## 2 MATERIAL AND METHODS

### 2.1 Ethics statement

The current study was performed with the approval of the Ethics Committee of Linyi People's Hospital and was conducted in strict adherence with the Declaration of Helsinki. All participants signed informed consent documentation. The animal study was conducted in line with an approved protocol provided by the Animal Care and Use Committee of Linyi People's Hospital in accordance with the National Institutes of Health guidelines.

### 2.2 Bioinformatics analysis

‘Limma’ package of ‘R’ software (http://www.bioconductor.org/packages/release/bioc/html/limma.html) and Gene Expression Omnibus database (GEO, https://www.ncbi.nlm.nih.gov/gds) were applied to screen the ESCC‐related microarray dataset GSE29001 to yield the relevant differential genes (|logFC| > 1, *P* < .05). There were 45 samples in GSE29001, 24 of which were from normal controls and 21 cases from ESCC patients. Gene Expression Profiling Interactive Analysis (GEPIA, http://gepia2.cancer‐pku.cn) was applied to analyse The Cancer Genome Atlas (TCGA, https://portal.gdc.cancer.gov) database. The top 500 genes correlated with the prognosis of EC were subsequently selected and converted to respective human transcription factor names using Cistrome (http://cistrome.org). Meanwhile, the downstream target genes of YAP1 were predicted using the Cistrome Cancer function. The microarray analysis results, the GEPIA analysis results and the transcription factor information acquired through Cistrome were plotted on a Venn diagram where the overlapping segment was indicative of the key transcription factors. StarBase (http://starbase.sysu.edu.cn) was employed to identify the expression tendencies of the key transcription factors. Survival curves of the transcription factors in ESCC were analysed by UALCAN (http://ualcan.path.uab.edu/index.html). The related genes of key factors were predicted through String (https://string‐db.org/) and the protein‐protein interaction (PPI) network were constructed using the Search Tool for the Retrieval of Interacting Genes (STRING, https://string‐db.org) and GeneMANIA (http://genemania.org). Cytoscape (http://www.cytoscape.org) was applied as the platform for visualizing molecular interaction networks. Potential downstream transcription factors were obtained by overlapping the results from STRING, GeneMANIA, and human transcription gene names and checked by GEPIA and hTFtarget (http://bioinfo.life.hust.edu.cn/hTFtarget#!). Previous literature was reviewed for further prediction of downstream regulation mechanism.

### 2.3 Tissue and cell culture

All the primary ESCC tissues as well as the adjacent tissues were collected via resection of specimens from 47 ESCC patients from Linyi People's Hospital between January 2012 and January 2014. Normal mucosa and anterior lesions were obtained and regarded as the controls. All patients were yet to undergo endoscopic mucosal resection, palliative resection, preoperative chemotherapy, or radiotherapy. All patients were confirmed to be free of simultaneous or multiple heterogeneous tumours on other organs. Follow‐up visits were performed until January 2019 and patients were monitored regularly. The average follow‐up time for surviving patients was 44 months (8 to 60 months). Besides, Kaplan‐Meier survive analysis was also performed (Table 1). Some patients were found to have little or no residual tumour, while other patients with small resections were excluded. Thus, certain types of the samples in some instances were missing from the patient.

**TABLE 1:** Patients information used for Kaplan‐Meier analysis

Time point (month)	Survival number of patients	Death number of patients	Origin
10	45	2	Linyi People's Hospital
20	39	8	Linyi People's Hospital
30	33	14	Linyi People's Hospital
40	28	19	Linyi People's Hospital
50	24	23	Linyi People's Hospital
60	17	30	Linyi People's Hospital

One oesophageal epithelial cell line (Het‐1A) and four EC cell lines (Eca‐109, SHEEC1, EC9706 and KYSE450) were purchased from China Center for Type Culture Collection and incubated in RPMI 1640 medium (Gbico, supplemented with 10% foetal bovine serum (FBS), penicillin and streptomycin (100 U/mL each) at 37℃ and 5% CO_2_.

### 2.4 Immunohistochemistry

The paraffin‐embedded tissues were sliced into 4‐μm‐thick sections, dewaxed in xylene, rehydrated in graded ethanol and subjected to antigen retrieval. The sections were then blocked in 10% normal serum and 1% bovine serum albumin (BSA) in TBS for 2 hours at room temperature. After washing with TBS buffer, the sections were incubated overnight at 4℃ with the primary antibodies: anti‐YAP (1:50, ab9572; Abcam), anti‐pc‐Jun (S63) (1:250, ab32385; Abcam), anti‐pc‐Jun (S73) (1:100, ab30620; Abcam) and anti‐phosphorylated JNK (1:50, #4668; Cell Signaling Technology). Endogenous peroxidase activity was then inactivated by adding 0.3% H_2_O_2_ (50 μL) and incubating at room temperature for 20 minutes. The sections were then added with polymer enhancer reagent (50 μL) for incubation at 37℃ for 30 minutes. Afterwards, the section was incubated with goat anti‐mouse IgG secondary antibody (50 μL, 1:2000, ab205718; Abcam) for 30 minutes at room temperature and then incubated with horseradish peroxidase (HRP)‐streptavidin reagent (Innova Biosciences) for 20 minutes, developed by freshly prepared 3,3‐diaminobenzidine (DAB, 100 μL or 2 drops). The sections were subsequently observed under a microscope for 3‐10 minutes. A brown colour was indicative of a positive case. The sections were then washed with distilled water, counter‐stained with haematoxylin, dehydrated in graded ethanol (75% ethanol, 95% ethanol and absolute ethanol), sealed with neutral resin, and observed and photographed under a microscope.

### 2.5 RNA extraction and quantification assay

The total RNA in the cells and tissues was isolated using a TRIzol Plus RNA Purification Kit (Invitrogen) in accordance with the user manual instructions. The RNA quality and concentration were then verified by the UV‐Vis spectrometer. mRNA was reversely transcribed to cDNA using the ImProm‐II™ Reverse Transcription System (Promega). Briefly, RNA (1 μg) was diluted in 12 μL ddH_2_O, denatured at 85℃ for 5 minutes, and placed on ice for further 5 minutes. Reverse transcription was performed by mixing RNA with Oligo dT (0.5 μL), random primer (0.5 μL), dNTP (10 mM, 2 μL), RNase inhibitor (0.5 μL), transcription buffer (5×, 4 μL), and M‐MLV reverse transcriptase (0.5 μL). cDNA was diluted to 50 ng/μL. Real‐time qPCR was performed using SYBR Premix Ex TaqTM II (Perfect Real Time) kit (DRR081; Takara). Glyceraldehyde‐3‐phosphate dehydrogenase (GAPDH) was regarded as the internal reference. The primer sequences for reverse transcription quantitative polymerase chain reaction (RT‐qPCR) are illustrated in Table 2.

**TABLE 2:** Primer sequences for RT‐qPCR

Genes	Forward (5′‐3′)	Reverse (5′‐3′)
YAP	GCATGATCTGCCCTAAGGC	TGACCGCCGAGTACACCAT
IRS2	AGCTCCCCCAAGTCTCCTAA	AGCCATCTCGGTGTAGTCAC
GAPDH	GGTGAAGGTCGGAGTCAACG	CCATGTAGTTGAGGTCAATGAAG

### 2.6 Nuclear and cytoplasmic extraction experiment

Nuclear and cytoplasmic extraction were conducted using NE‐PER™ Nuclear and Cytoplasmic Extraction Reagents (Thermo Fisher). Quantitative analysis of nuclear or cytoplasmic protein was performed by Western blot.

### 2.7 Western blot assay

Tissue and cell total protein were extracted using PMSF or RIPA lysis buffer at 4℃ for 30 minutes, followed by centrifugation at 8000 *g* for 10 minutes according to the user manual. After the supernatant had been collected, protein concentration was determined and normalized using bicinchoninic acid (BCA) kit (P0012; Beyotime Biotechnology). The proteins were subsequently separated via sodium dodecyl sulphate‐polyacrylamide gel electrophoresis (SDS‐PAGE) and transferred onto polyvinylidene fluoride (PVDF) membrane (Millipore). Membrane blockade was performed using 5% skimmed milk powder for 1 hour at room temperature and then incubated at 4℃ overnight with diluted primary antibodies: rabbit anti‐YAP (1:5000, ab52771; Abcam), anti‐c‐Jun (1:1000, ab31419; Abcam), anti‐pc‐Jun (S63) (1:10000, ab32385; Abcam), anti‐pc‐Jun (S73) (1:1000, ab30620; Abcam), anti‐phosphorylated JNK1/2 (1:1000, #4668; Cell Signaling Technology), anti‐JNK (1:1000, #9252; Cell Signaling Technology), and GAPDH (1:10 000, ab8245; Abcam). Following incubation, the membrane was washed three times using TBST buffer for 10 minutes. HRP labelled goat anti‐rabbit IgG (1:10 000, ab205718; Abcam), goat anti‐mouse IgG (1:10 000, ab205719; Abcam) was added for an additional round of 1‐hour incubation at room temperature. The immunoblots were visualized with enhanced chemiluminescence reagents (WBKLS0100; Millipore). The images were captured and analysed by ImageJ 1.48u (Bio‐Rad).

### 2.8 Transwell invasion assay

Tumour invasion assays were performed using Transwell chambers (8 μM pore size; Corning Incorporated). The upper chamber was treated with Matrigel™ basement membrane matrix (BD Biosciences). Following transfected with either HTR8/SVneo or the negative control for 48 hours, the cells were resuspended in serum‐free medium and added to the upper chambers. Complete medium containing 20% FBS was added to the bottom wells of the chambers. The chambers were incubated at 37℃, 5% CO_2_ for 48 hours. Next, to determine the number of invaded cells, the lower surfaces of the filters were fixed with 4% paraformaldehyde solution and stained with crystal violet. Images from five different fields were captured from each membrane after which the number of invasive cells was counted. The mean value was obtained from the triplicate assays of each experiment.

### 2.9 Colony formation assay

The cells were trypsinized and incubated into 6‐well plates (2000 cells/100 μL each well) at 37 °C, with the medium changed at regular 2 day intervals. Following removal of the medium, the cells were washed twice with PBS and fixed with 5% paraformaldehyde for 30 minutes. The cells were then incubated with 0.1% crystal violet solution (Solarbio) for 20 minutes at room temperature. The number of clones was counted under microscope.

### 2.10 Assessment of viability by cell counting kit (CCK)‐8 assay

The cells were pre‐cultured in 96‐well plate for 24 hours at 37℃ with 5% CO_2_ and transfected with si‐RNA. Cell viability was assessed using a cell counting kit (CCK)‐8 kit (Beyotime Biotechnology) after 0 hour, 24 hours, 48 hours and 72 hours of transfection. The cells were incubated for a further 4 hours prior to recording the absorbance at a wavelength of 450 nm on a microplate reader.

### 2.11 Co‐immunoprecipitation assay

The cells were lysed in buffer containing Tris‐HCl (50 mM, pH 7.5), NaCl (150 mM), Nonidet P‐40 (1%), sodium deoxycholate (0.5%), and proteinase (1%; Sigma‐Aldrich) and subjected to sonication. After the cell lysis had been centrifuged, the supernatant was incubated overnight at 4℃ with anti‐YAP, anti‐TEAD and Protein G Plus/Protein A Agarose beads (CalBiochem) or IgG beads of the same type (Sigma‐Aldrich). The beads were subsequently washed six times using a lysis buffer and analysed by Western blot.

### 2.12 Chromatin immunoprecipitation assay

The cells were fixed using 1% formaldehyde and sheared by sonication. The antibody was added and mixed with the promoter. The antibody‐promoter complex was precipitated through the addition of Protein A Agarose/Salmon Sperm DNA. Nonspecific binding was washed away prior to eluting and de‐crosslinking the promoter complex. The promoter fragments were purified and employed as the RT‐qPCR template.

### 2.13 Assessment of cell sphere formation

The cells were transfected with si‐YAP or control si‐RNA. After 48 hours, a total of 2500 cells were cultured in serum‐free DMEM/F12 medium (Gibco) supplemented with B‐27 (1:50; Invitrogen), and fibroblast growth factors (FGF, 20 ng/mL; R&D Systems) in the ultra‐low attachment plates (Corning). After 10‐14 days, the sphere was fixed using methanol. The average number of primary spheres and their radius were analysed by ImageJ. Only the clusters with a diameter of >50 μm were counted.

### 2.14 Assessment of tumour formation in nude mice

A total of 30 BALB/c male nude mice aged 4‐ to 5‐week‐old were purchase from Beijing Vital River Laboratory Animal Technology. The EC9706 cells were infected with the following lentiviruses: negative control (sh‐NC + e‐NC), YAP silence (sh‐YAP + oe‐NC), YAP silence with IRS2 overexpression (sh‐YAP + oe‐IRS2). Cells at the logarithmic growth phase were then subcutaneously inoculated into the back of the mice. Tumour volume was measured using a Vernier caliper, with the calculation performed using the formula: length × width^2^ × π/6. The mice were euthanized following the completion of the experiment. The tumours were subsequently removed, weighed, fixed and paraffin‐embedded.

### 2.15 Statistical analysis

All statistical data analyses were performed using SPSS 19.0 (IBM Corp. Armonk, NY, USA). Measurement data were expressed as the mean ± standard deviation. Data comparisons between two groups were analysed via paired *t* test or independent *t* test. Statistical analysis in relation to time‐based measurements within each group was realized using repeated‐measures analysis of variance (ANOVA) with Tukey's post hoc test. Comparisons among multiple groups were conducted using one‐way ANOVA with Tukey's post hoc test. All experiments were conducted in triplicate and independently. Pearson's correlation coefficient was used to analyse the relationship between YAP and IRS2. The survival rate of 47 patients was calculated using the Kaplan‐Meier method. The Log‐rank test was used to detect the difference in survival. A value of *P* < .05 was considered to be indicative of statistically significant difference.

## 3 RESULTS

### 3.1 YAP is overexpressed in ESCC tissues and cells

**FIGURE 1:**
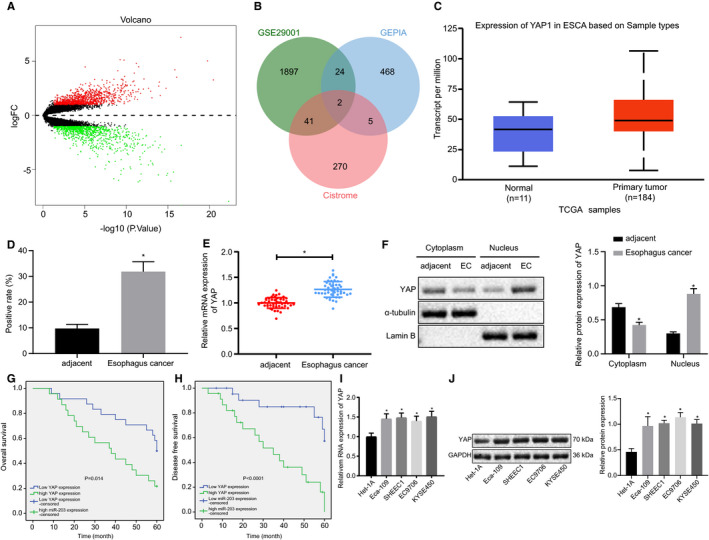
YAP is overexpressed in ESCC tissues and cells. (A) The volcanic plot of gene regulation analysis in microarray data set GSE29001. Genes significantly up‐regulated are highlighted as red dots while significantly down‐regulated are green; (B) Venn diagram of significant differential gene set obtained from microarray data set GSE29001, first 500 genes related to ESCC survival, and human transcription factors. Overlapping genes are FOS and YAP (YAP1); (C) Box plot of YAP expression in EC analysed by StarBase; (D) Immunohistochemical staining of YAP in EC, five fields were randomly observed, n = 47; (E) RT‐qPCR assay of YAP expression (N = 47); (F) Nuclear and cytoplasmic extraction experiment for detecting YAP expression; (G) Kaplan‐Meier curve of overall survival with high YAP expression; (H) Kaplan‐Meier curve of disease‐free survival with high YAP expression; (I) RT‐qPCR quantification of YAP level in an oesophageal epithelial cell line (Het‐1A) and four EC cell lines (Eca‐109, SHEEC1, EC9706 and KYSE450). (J) Western blot quantification of YAP level in an oesophageal epithelial cell line (Het‐1A) and four EC cell lines (Eca‐109, SHEEC1, EC9706 and KYSE450). Measurement data are presented as mean ± standard deviation. Comparisons in panel B were performed by paired *t* test, *, *P* < .05, vs adjacent tissues, or *, *P* < .05, vs Het‐1A cell. Comparisons among multiple groups were performed by one‐way analysis of variance (ANOVA) with Tukey's post hoc test. All experiments were conducted independently in triplicate. ESCC, squamous cell carcinoma; YAP, Yes‐associated protein

The differential analysis of GSE29001 in the GEO database by R language revealed 1964 differentially expressed genes, among which 1047 were up‐regulation genes while 917 were down‐regulation genes (Figure 1A). GEPIA was employed to analyse the genes in relation to EC survival in the TCGA database, with the top 500 genes subsequently identified. To further elucidate the mechanisms of transcription factors in EC, the human transcriptional factor names were subsequently obtained from Cistrome. The analysis results obtained in connection with GSE29001, GEPIA and Cistrome were intersected, with FOS and YAP (YAP1) identified as essential transcriptional factors associated with EC survival (Figure 1B). The TCGA database was analysed by UALCAN, and we identified the overexpression of YAP in EC (Figure 1C).

Primary ESCC tissues and adjacent tissues from 47 patients were evaluated for YAP expression detection. Immunohistochemistry staining (Figure 1D and Figure S1) indicated high levels of YAP in ESCC tissues, and that YAP was mainly localized in nuclear. RT‐qPCR confirmed an increased level of YAP in ESCC tissues (Figure 1E). Next, to further investigate the difference of YAP expression inside the cells, the nuclear and cytoplasmic extraction experiment were performed, followed by Western blot assay. As depicted in Figure 1F, the level of YAP was significantly elevated in cell nuclear, which was consistent with the immunohistochemistry image findings. Moreover, the Kaplan‐Meier curves highlighted that high levels of YAP expression were associated with a decrease in both disease‐free survival and overall survival (Figure 1G,H). Further, RT‐qPCR and Western blot quantification of YAP level in an oesophageal epithelial cell line (Het‐1A) and four EC cell lines (Eca‐109, SHEEC1, EC9706 and KYSE450) were indicative of significantly elevated levels of YAP in the EC cell (Figure 1I, J). The above results provided evidence of high expression of YAP in ESCC tissues and cells.

### 3.2 Silencing YAP inhibits the proliferation, invasion and sphere formation of EC cells

**FIGURE 2:**
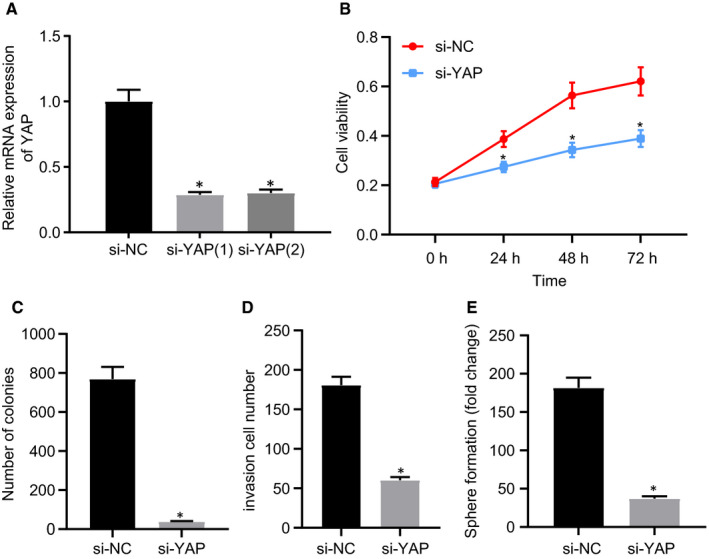
Knocking down YAP inhibits the proliferation, invasion and sphere formation of EC9706 cells. (A) The efficiency of si‐YAP knocking down checked by RT‐qPCR; (B) CCK‐8 assay of cell viability in the si‐YAP group and si‐NC group; (C) Clonogenic assay of the munber of cell clones; (D) Transwell invasion assays to determine the carcinoma invisibility; (E) Tumour sphere‐forming from the si‐NC group and the si‐YAP group. Measurement data are presented as mean ± standard deviation. Data comparisons between two groups were analysed by independent *t* test. Statistical analysis in relation to time‐based measurements within each group was realized using repeated‐measures ANOVA with Tukey's post hoc test. *, *P* < .05, vs si‐NC group. All experiments were conducted independently in triplicate. YAP, Yes‐associated protein

Two si‐RNAs were specifically designed for YAP gene silencing and transfected into EC9706 cells. Figure 2A illustrates the YAP expression RT‐qPCR findings. No.2 si‐RNA was selected for subsequent experiments due to its superior silencing efficiency. After loss‐of function, evidence was obtained indicating that when YAP was silenced, cell viability, the number of formed clones, invasiveness and sphere‐forming capacity reduced significantly (Figure 2B‐E and Figure S2). We repeated the experiments in Eca‐109 cells, with similar results to those in EC9706 cells obtained (Figure S3). The aforementioned observations led to the conclusion that YAP silencing alleviated EC activity.

### 3.3 YAP activates JNK/c‐Jun pathway by binding to TEAD

**FIGURE 3:**
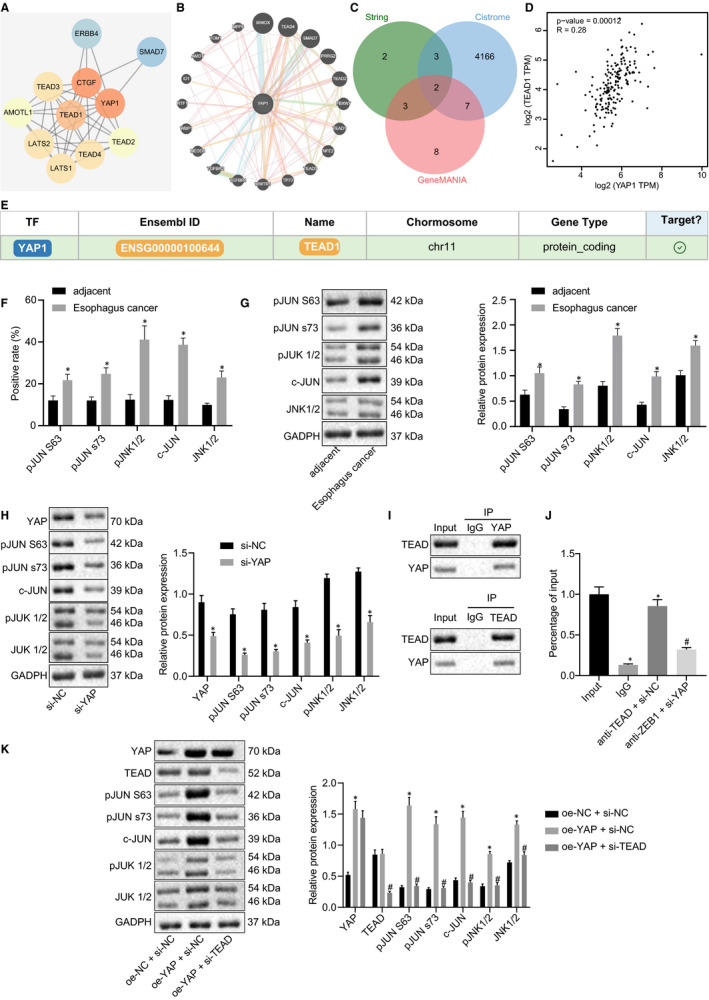
YAP binds to TEAD to activate the JNK/c‐Jun pathway in EC cells. (A) PPI network graph of YAP and related genes constructed by STRING. Colour of nodes from red to blue indicates the transition from hyper‐core level to non‐core level genes; (B) PPI network graph of YAP and related genes constructed by GeneMANIA. The size of the node indicates the score in the PPI network, the higher the score the larger the node. (C) Venn diagram of gene sets related to YAP predicted from STRING and GeneMANIA, and human transcription factors. Overlapped area indicates SMAD7 and TEAD1; (D) Scatter plot of correlation analysis of YAP and TEAD1 obtained from GEPIA (*r* = .28, P = 1.2E‐04); (E) Targeting relationship of YAP and TEAD predicted by hTFtarget, the last column shows a targeting relationship between the two predicted by website; (F) Immunohistochemistry of ESCC tissues and adjacent tissues with pc‐Jun (S63), pc‐Jun (S73) and phosphorylated JNK1/2 staining (N = 47); (G) Western blot assay to determine the expression of pc‐Jun (S63), pc‐Jun (S73), and phosphorylated JNK1/2 (N = 47); (H) Western blot assay to determine the expression of pc‐Jun (S63), pc‐Jun (S73), c‐Jun, phosphorylated JNK1/2 and JNK1/2 after knocking down YAP in EC9706 cells; (I) Co‐immunoprecipitation assay of YAP and TEAD; (J) ChIP‐PCR assay to verify TEAD binds c‐Jun promoter; (K) Western blot assay to determine the expression of pc‐Jun (S63), pc‐Jun (S73), c‐Jun, phosphorylated JNK1/2 and JNK1/2 after transfection of oe‐YAP and oe‐YAP + si‐TEAD in EC9706 cells. GAPDH served as the internal reference in all the Western blot assays. Measurement data are presented as mean ± standard deviation. Data comparisons between the two groups were analysed by independent pair *t* test. Comparisons among multiple groups were performed by one‐way analysis of variance (ANOVA) with Tukey's post hoc test. All experiments were conducted independently in triplicate. *, *P* < .05, vs adjacent group, si‐NC group, IgG group, or oe‐NC + si‐NC group. ^#^, *P* < .05, vs oe‐YAP + si‐NC group, or oe‐YAP + si‐TEAD group. ESCC, squamous cell carcinoma; YAP, Yes‐associated protein

In order to elucidate the downstream mechanism of YAP, we predicted ten YAP (YAP1)‐related genes using STRING and twenty genes using GeneMANIA. In Figure 3A,B, we obtained the related genes of YAP1 through STRING and GeneMANIA databases and constructed the PPI network of the YAP‐related genes, respectively. Next, by taking the intersection of the obtained related genes with the downstream genes of YAP predicted by Cistrome, we finally obtained 2 candidate genes, SMAD7 and TEAD1 (Figure 3C). Existing literature has suggested that YAP influences EC occurrence by binding to TEAD (TEAD1),^6^ which encouraged us to analyse the expression of TEAD in EC. As indicated by GEPIA analysis in Figure 3D, YAP and TEAD (TEAD1) revealed the existence of a distinct link. Besides, the targeting relationship between YAP and TEAD was solidified by analysing the transcriptional gene database hTFtarget (Figure 3E). Thus, we chose TEAD for subsequent research instead of SMAD7.

Considering that the JNK/c‐Jun pathway is over‐activated in more than 80% oesophageal adenocarcinoma specimens,^25^ and YAP‐TEAD can monitor the progression of basal cell carcinoma via JNK/c‐Jun pathway,^18^ we set out to investigate the same pathway in EC. The expression of pc‐Jun (S63), pc‐Jun (S73), and phosphorylated JNK1/2 in primary ESCC tissues and adjacent tissues were identified using immunohistochemistry methods. As depicted in Figure 3F and Figure S4A, both the expression and the nuclear localization of the three above proteins exhibited increases in ESCC tissues. Quantification from Western blot assay in ESCC tissues provided evidence validating this observation (Figure 3G). In the event of YAP silencing with si‐RNA, expression of pc‐Jun (S63), pc‐Jun (S73), c‐Jun, phosphorylated JNK1/2 and JNK1/2 was reduced in EC9706 cells, demonstrated by Western blot assay (Figure 3H).

Previous reports have suggested that in basal cell carcinoma, the c‐Jun promoter and enhancer contain not only a TEAD binding site but also multiple AP1 recognition sites which can be bound by c‐Jun and regulate c‐Jun expression.^18^ Besides, genome‐wide correlation of YAP/TAZ/TEAD with AP‐1 promotes tumour growth.^11^ Therefore, the relationship between YAP and JNK/c‐Jun pathway in EC was examined. Initially, evidence was obtained highlighting interactions between YAP and TEAD based on the co‐immunoprecipitation assay results (Figure 3I). Next, data were obtained showcasing that TEAD bound to c‐Jun promoters using Chromatin immunoprecipitation (ChIP)‐PCR. The enrichment got lessened when YAP was silenced (Figure 3J). Finally, Western blot assay displayed that the overexpression of YAP triggered an increase in the expression of pc‐Jun (S63), pc‐Jun (S73), c‐Jun, phosphorylated JNK1/2 and JNK1/2 in EC9706 cells, which was rescued following TEAD silencing in the YAP‐overexpressed EC9706 cells (Figure 3K). Taken together, the aforementioned findings suggested that YAP interacted with TEAD, and that YAP activated the JNK/c‐Jun pathway by binding to TEAD.

### 3.4 YAP‐TEAD activates IRS2 via JNK/c‐Jun pathway

**FIGURE 4:**
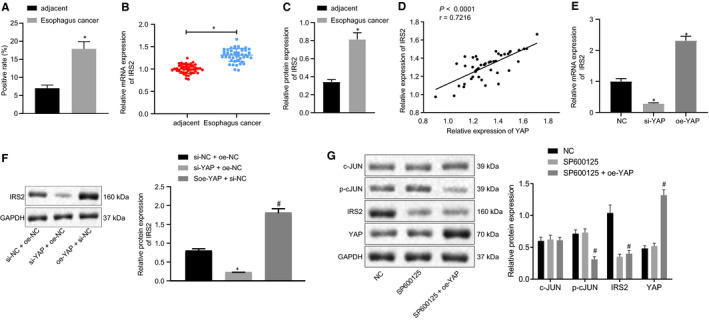
YAP‐TEAD actives IRS2 expression via the JNK/c‐Jun pathway in EC cells. (A) Immunohistochemistry staining of IRS2 in ESCC tissues and adjacent tissues; (B) RT‐qPCR to determine IRS2 expression (N = 47); (C) Western blot assay to determine IRS2 expression (N = 47); (D) Pearson analysis of expression correlation of YAP and IRS2; (E) RT‐qPCR to determine IRS2 expression after transfection of si‐YAP or oe‐YAP into EC9706 cells; (F) Western blot assay to determine IRS2 expression after transfection of si‐YAP or oe‐YAP into EC9706 cells; (G) Western blot assay to determine IRS2 expression after JNK treatment of inhibitor SP600125 or overexpressing YAP. Measurement data are presented as mean ± standard deviation. Data comparisons in panel B were analysed by paired *t* test. Data comparisons between the two groups were analysed by independent pair *t* test. Comparisons among multiple groups were performed by one‐way analysis of variance (ANOVA) with Tukey's post hoc test. All experiments were conducted independently in triplicate. *, *P* < .05, vs adjacent tissues, negative control group; ^#^, *P* < .05, vs SP600125‐treated group. ESCC, squamous cell carcinoma; YAP, Yes‐associated protein

IRS2 is reported to be the downstream target gene of the JNK pathway.^26^ With immunohistochemistry staining, the data obtained indicated that the expression of IRS2 was markedly elevated in the ESCC tissues (Figure 4A and Figure S4B). The up‐regulation of IRS2 was confirmed by data obtained from RT‐qPCR and Western blot assay (Figure 4B,C and Figure S4C), while a positive correlation between the expression of IRS2 and YAP expression was uncovered (Figure 4D). Thereafter, we set out to evaluate whether the expression of IRS2 was regulated by YAP. Following the overexpression of YAP in EC cells, RT‐qPCR and Western blot assay results suggested that the expression of IRS2 was elevated (Figure 4E). Correspondingly, silencing YAP suppressed IRS2 expression (Figure 4F). We previously found that c‐Jun and the phosphorylated c‐Jun (pc‐Jun) were up‐regulated in the YAP‐overexpressed cells. In line without predictions, the JNK pathway inhibitor SP600125 deceased JNK‐mediated c‐Jun phosphorylation and IRS2 expression, thus the overexpression of YAP failed to upregulate IRS2 expression (Figure 4G). These findings suggested IRS2 expression was regulated by the JNK/c‐Jun pathway. Suppressing the JNK/c‐Jun pathway restrained IRS2, which could not be rescued by YAP overexpression. In short, YAP increased IRS2 expression via the JNK/c‐Jun pathway.

### 3.5 YAP elevates IRS2 to induce and deteriorate EC

**FIGURE 5:**
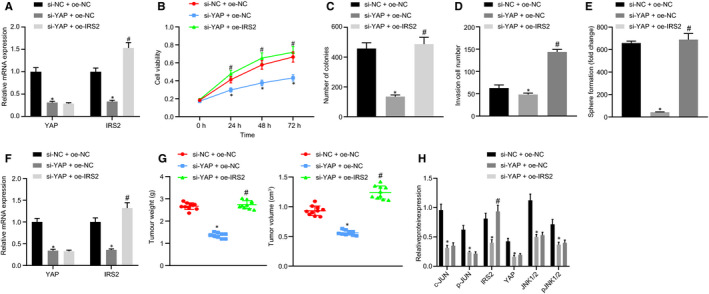
YAP induces and deteriorates ESCC by activating IRS2. (A) Expression of YAP and IRS2 in EC9706 cells after treatment of si‐YAP or oe‐IRS2 detected by RT‐qPCR; (B) CCK‐8 assay for cell viability after treatment of si‐YAP or oe‐IRS2; (C) Clonogenic experiments results after treatment of si‐YAP or oe‐IRS2; (D) Cell invasion assay to determine cell invasiveness after treatment of si‐YAP or oe‐IRS2; (E) Quantification of sphere formation after treatment of si‐YAP or oe‐IRS2; (F) RT‐qPCR quantification of YAP and IRS2 expression in xenografted nude mice bearing si‐YAP or oe‐IRS2 (N = 10); (G) Tumour weight and volume in nude mice; (H) Western blot analysis of YAP, JNK/p‐JNK, c‐Jun/p‐c‐Jun, and IRS2 expression in tumours of mice bearing si‐YAP or oe‐IRS2 (N = 10). Measurement data were reported as mean ± standard deviation. Comparisons among multiple groups were performed by one‐way analysis of variance (ANOVA) with Tukey's post hoc test. Statistical analysis in relation to time‐based measurements within each group was realized using repeated‐measures ANOVA with Tukey's post hoc test. All experiments were conducted independently in triplicate. *, *P* < .05, vs si‐NC + oe‐NC group. ^#^, *P* < .05, vs si‐YAP + oe‐IRS2 group. ESCC, squamous cell carcinoma; YAP, Yes‐associated protein

Next, we set out to ascertain whether YAP deteriorates ESCC by up‐regulating IRS2 in EC. In the YAP‐silenced EC9706 cell, IRS2 expression was decreased. Compared to treatment with si‐YAP alone, silencing YAP and overexpressing IRS2 simultaneously increased the IRS2 level but kept YAP expression unchanged (Figure 5A). Functional experiments conducted demonstrated that IRS2 overexpression reversed the inhibited cell proliferation, invasiveness and sphere formation in YAP‐silenced cells (Figure 5B‐E). The above observations suggested that YAP deteriorated EC by activating IRS2 in vitro.

Finally, we investigated tumour formation in nude mice. EC9706 cells transfected with lentivirus pf sh‐YAP + oe‐NC or sh‐YAP + oe‐IRS2 were subcutaneously inoculated into the back of the mice. The expression of YAP and IRS2 in xenografted nude mice was quantified by RT‐qPCR, as depicted in Figure 5F, with consistent results obtained from the cell experiments. Tumorigenesis after EC9706 inoculation in mice revealed that silencing YAP inhibited the progression of EC and reduced tumour size and weight, which was neutralized following the overexpression of IRS2 (Figure 5G). Western blot analysis further revealed that silencing YAP markedly decreased the expression of YAP, JNK/p‐JNK, c‐Jun/p‐c‐Jun and IRS2, while overexpression of IRS2 significantly increased IRS2 expression but had no effect on other proteins (Figure 5H). Collectively, the aforementioned results indicated that YAP deteriorated EC via activation of IRS2 both in vivo and in vitro.

## 4 DISCUSSION

**FIGURE 6:**
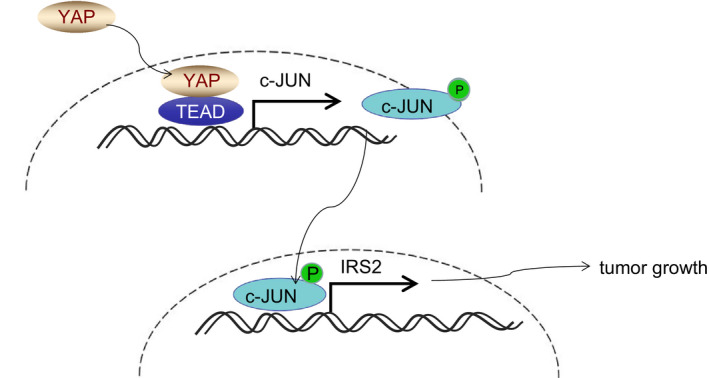
Schematic diagram of YAP/TEAD/c‐Jun/IRS2. YAP positively regulates IRS2 expression, thus inducing and deteriorating ESCC via the JNK/c‐Jun pathway. ESCC, squamous cell carcinoma; YAP, Yes‐associated protein

Oesophageal cancer remains a significant cause of cancer‐related mortality worldwide. The current study set out to elucidate a new regulatory axis, YAP‐TEAD/JNK/c‐Jun/IRS2, which was proven to be a notable factor in the occurrence and progression of EC. The YAP‐TEAD complex was enriched in the c‐Jun promoter in addition to stimulating its phosphorylation. Our data indicated that pc‐Jun up‐regulated IRS2 while deteriorating ESCC (Figure 6).

Other than common external carcinogens, genetic transcription factors represent significant contributors to ESCC occurrence and progression.^2^, ^27^ Through the exploration and mining of the bioinformatic database, YAP was found to be related to EC survival. We subsequently evaluated the effects associated with YAP on proliferation, invasion and sphere formation of EC cells. Key observations made during our study were largely consistent with previous research attesting that YAP advances ESCC^28^ while inhibition of Hippo pathway results decreased cell proliferation.^29^ Previous studies have emphasized the link between YAP and ESCC progression whereby YAP was overexpressed in EC tissues.^26^, ^30^, ^31^ YAP has been reported to influence the occurrence of EC by means of binding to TEAD (TEAD1),^6^ while the interaction between TEAD and YAP has been shown to contribute to the development of EC,^12^ providing insight into the role of YAP/TEAD signalling in the EC deterioration.

In light of the fact that the c‐Jun promoter and enhancer contain TEAD binding sites, we investigated the role of c‐Jun in our pathway. In ESCC, c‐Jun activates the promoters of differentiation‐associated genes,^32^ while phosphorylated c‐Jun (pc‐Jun) accounts for cisplatin resistance,^33^ apoptosis inhibition^34^ and radiosensitivity acquisition.^35^ Previous studies have speculated that c‐Jun may be destructive or constructive, depending on the JNK1 or JNK2 pathway, in relation to EC treatment. Previous literature has implicated c‐Jun in the modulation of genes associated with differentiation in ESCC.^32^ Activation of JNK1 leads to Bcl‐2 phosphorylation and autophagy in primary effusion lymphoma.^36^ Wu et al^37^ suggested that JNK1 crosstalks with STAT3 in RAW264.7 macrophage cells. In light of the evidence regarding c‐Jun as a substrate of JNK, we excluded other transcription factors which may potentially contribute to this pathway.

Interestingly, the phosphorylation levels of JNK1/2 and JNK1/2 were diminished in the event of YAP knock down, indicating that the expression and phosphorylation of JNK were regulated by YAP and its transcriptional partner. It is also possible that JNK is feedback monitored by pc‐Jun or other factors in the YAP downstream pathway. Several ubiquitin ligases, including FBXO31^38^ and RAD18,^39^ have been demonstrated to possess the capacity to modulate the phosphorylation levels of JNK in ESCC; hence, the current study identified the upstream transcription factors and kinases of JNK1/2.

A positive correlation has been suggested between YAP‐TEAD and IRS2 expression in the context of liver cancer^21^ as well as non‐small cell lung cancer.^19^ However, direct evidence demonstrating that pc‐Jun binds to IRS2 promoter remains elusive. The insulin receptor substrate (IRS) family consists of at least four members, IRS1, IRS2, IRS3 and IRS4.^40^ A study with IRS2‐knockout mice highlighted the critical role of IRS2 in cell growth and hormone secretion.^41^ In ESCC, IRS1 and IRS2 are overexpressed and promote cell proliferation.^23^, ^42^ Consistently, IRS2 has been shown to deteriorate EC in our study.

In conclusion, the key observations of our study provide evidence of YAP‐TEAD‐activated JNK/c‐Jun pathway to up‐regulate IRS2, thus promoting cell proliferation and invasion of EC cells. The downstream genes of YAP could be diverse; hence, further investigation is required.

## CONFLICT OF INTEREST

The authors declare that they have no competing interests

## AUTHOR CONTRIBUTIONS

**Xiangming Xu:** Conceptualization (equal); Writing‐original draft (equal). **Jiao Nie:** Data curation (equal); Formal analysis (equal). **Lin Lu:** Investigation (equal); Visualization (equal). **Chao Du:** Software (equal); Supervision (equal). **Fansheng Meng:** Project administration (equal); Resources (equal). **Duannuo Song:** Methodology (equal); Writing‐review & editing (equal).

## Supporting information

Figure S1

Figure S2

Figure S3

Figure S4

## Data Availability

Research data are not shared.
